# Supplementary material for: O-Doping Configurations Reduce the Adsorption Energy Barrier of K-Ions to Improve the Electrochemical Performance of Biomass-Derived Carbon
Source: Micromachines (Basel). 2022 May 22;13(5):806. doi: 10.3390/mi13050806 (PMC9143850; doi:10.3390/mi13050806)
Supplement: Supplementary file 1 [file micromachines-13-00806-s001.zip › micromachines-1731457-supplementary.pdf]

O-doping configurations reduce the adsorption energy barrier of K-ion to improve the electrochemical performance of biomass-derived carbon

Kai Zhao [1,†], Changdong Chen [2,†], Ming La[3,*], and Chenghao Yang [2,]**

[1] College of Information Engineering, Pingdingshan University, Pingdingshan, Henan 467000, China.

[2] School of Enviornment and Energy, South China University of Technology, Guangzhou 510006, China.

[3] College of Chemistry and Environmental Engineering, Pingdingshan University, Pingdingshan, Henan 467000, China.

[†] The authors contributed equally to this work.

***** Correspondence: laming82@126.com (M. La); esyangc@scut.edu.cn (C. Yang).

1. Experiment

1.1 Material Preparation

The oranges were bought from supermarkets in Hebei province, China. In order to prepare OPDC, carbonization and airflow-annealing processes are necessary. For the typical synthesis process, the rinsed orange peel was dried, ground and placed in the tubular furnace at 1000 °C for 2 h (N_2 atmosphere). The carbonized product was then further annealed at 350 °C for 3 h (air atmosphere). Finally, the activated product was soaked in 3M HCl to remove impurities, and OPDC was obtained after washing and drying.

1.2 Material Characterization

For OPDC, the macromorphology was observed by TESCAN VEGA3 scanning electron microscope (SEM, Czech Republic), the microstructure was obtained by FEI Talos 200S high resolution transmission electron microscope (HRTEM, Netherlands), the crystal structure was characterized by Mini Flex600 X-ray diffraction analyzer (XRD, Japan). The HORIBA LabRAM HR Evolution Raman spectrometer (Raman, France) was used to determine information about molecular vibration or rotation. Micromeritics APSP 2460 specific surface area and porosity analyzer (USA) studied the N_2 adsorption/desorption by OPDC and calculated the specific surface area using the Brunauer/Emmett/Teller (BET) model. The states of chemical bonds or functional groups in molecules were analyzed by Thermo Scientific Nicolet IS50 fourier transform infrared spectrometer (FTIR, USA). The elemental valence state information on the material surface was characterized using Thermo Fisher Scientific K-Alpha X-ray photoelectron spectrometer (XPS, USA).

1.3 Electrochemical Performance Test

According to the mass ratio of 8:1:1, OPDC was evenly mixed with super p, polymerized styrene butadiene rubber and carboxymethyl cellulose, and water was used as the solvent to disperse into slurry, which was coated on the current collector and rolled to the appropriate thickness after vacuum drying. The OPDC anode, glass fiber separator and metal K cathode were assembled into 2025 half cell, and the electrochemical performance under different conditions were tested. The main components of the electrolyte solution are 0.8 M

KPF_6-ethylene carbonate/diethyl carbonate (1:1 in volume).

The specific charge/discharge capacity, cycle performance, rate characteristics and galvanostatic intermittent titration technique (GITT) tests were performed on the LANGHE CT2001A battery test system (China). The GITT curve was obtained by an activation cycle with a pulse current of 50 mA/g for 0.5 h, and then stationary for 2 h. The cyclic voltammetry (CV) characteristics of the same scanning rate (0.1 mV/s) and various scanning rates (0.3, 0.5, 0.7 and 1 mV/s) were measured by Chenhua CHI760E electrochemical workstation (China). At the same time, the electrochemical workstation was also used to test electrochemical impedance spectroscopy (EIS) in the frequency range of 10^5-10^{-2} Hz. All electrochemical tests were performed at room temperature with a voltage range of 3.0-0.01 V (vs. K^+/K).

1.4 Theoretical Calculation

Spin-polarized DFT was performed by using the Vienna Ab initio Simulation Package (VASP).[1] The generalzied gradient approximation proposed by Perdew, Burke, and Ernzerhof is selected for the exchange-correlation potential.[2] The cut-off energy for plane wave is set to 400 eV. The energy convergence and atomic forces were set to 10^{-5} eV and 0.02 eV/Å. The Brillouin zone is set to Gamma-centered point 3×3×1 for the structure optimization. The vacuum region was set as 15 Å in z direction to prevent the interaction between two adjacent surfaces.

The calculate adsorption energies (E_{ads}) of K-ion on surface according to the equation:

$$E_{ads} = E_{total}-E_{surf}-E_{K}$$

E_{total} and E_{surf} are the total energies of surface with and without K-ion adsorption, E_K is the energy for the bulk phase.

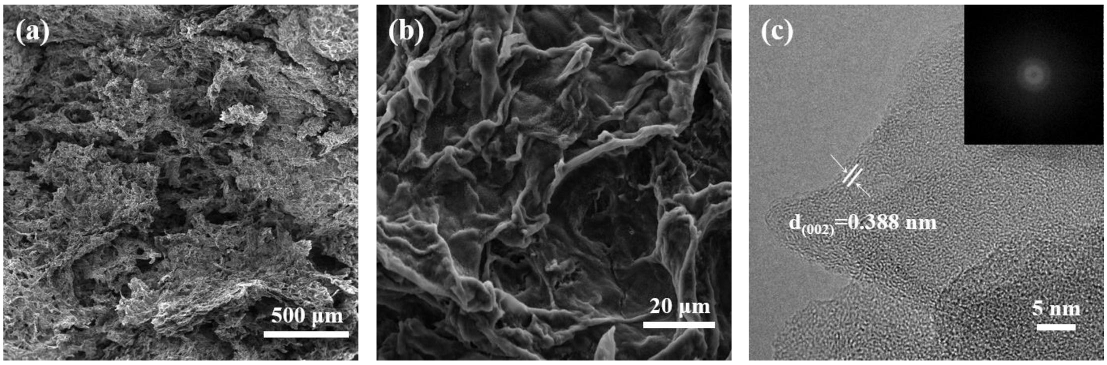

Figure S1. SEM images of OPDC with different magnification: (a) ×0.1 k and (b) ×2.0 k. (c) HRTEM and fast Fourier transform (FFT) images of OPDC.

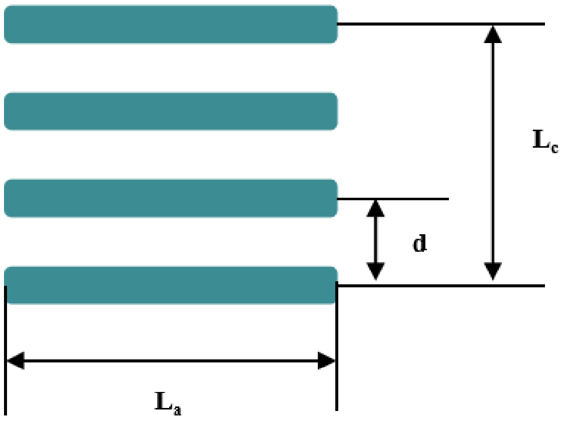

Figure S2. Illustration of the calculation for domain thickness.

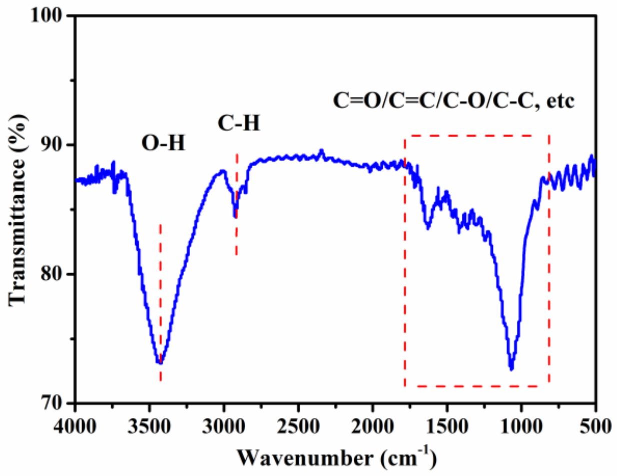

Figure S3. FTIR of OPDC.

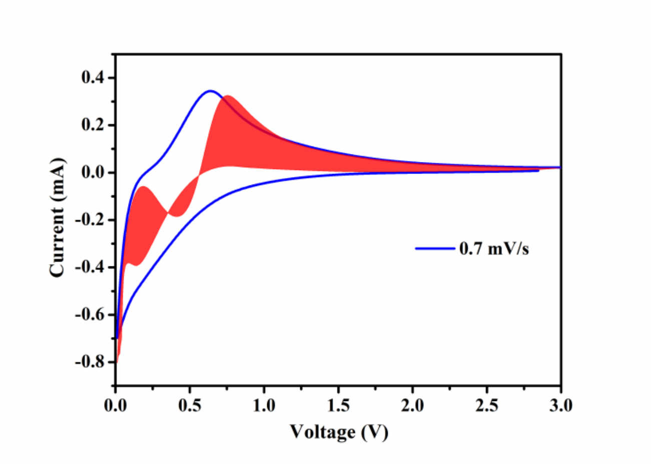

Figure S4. Contribution ratio of the surface adsorption behavior at the scan rate of 0.7 mV/s.

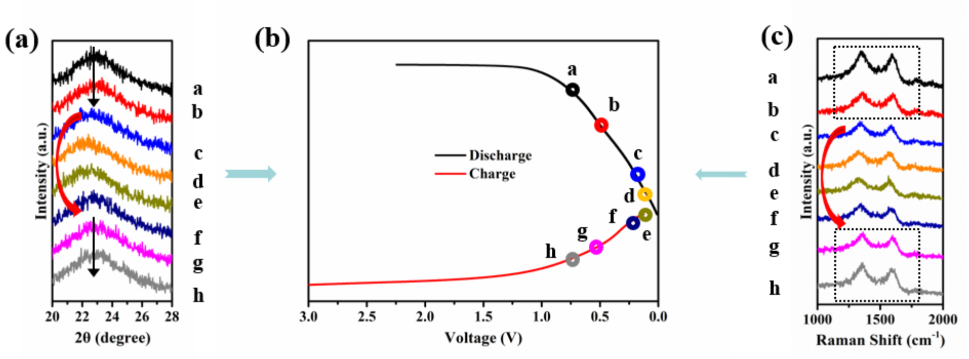

Figure S5. (a) XRD patterns of the OPDC electrode during discharging and charging, (b) The corresponding galvanostatic charge/discharge profile, and (c) Raman spectra of the OPDC electrode during discharging and charging.

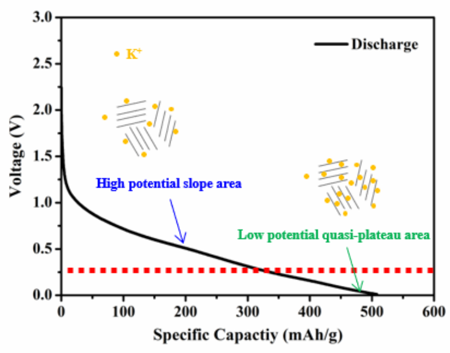

Figure S6. Schematic diagram of the potassium-ion storage mechanism in the OPDC electrode.

Table S1. R_{SEI} and R_{ct} simulated from equivalent circuit analysis at different cycles.

Cycles	1st	10th	50th	150th
Ro	8.14 Ω	7.64 Ω	6.16 Ω	6.90 Ω
RSEI	46.78 Ω	45.48 Ω	49.07 Ω	56.21 Ω
Rct	1406.00 Ω	685.21 Ω	880.52 Ω	2088.04 Ω

Table S2. Comparison of the electrochemical performance between OPDC and partial biomass derived carbon.

Precursor	Doped atom	Initial Coulombic efficiency	Performance	Ref.
Tremella	N	24.3%	290.4 mAh/g at 100 mA/g<br>119.7 mAh/g at 2000 mA/g	[3]
Dandelion seed	N	46.9%	168 mAh/g at 50 mA/g<br>49 mAh/g at 1000 mA/g	[4]
Maize straw	N	39.9%	123 mAh/g at 50 mA/g<br>30 mAh/g at 1000 mA/g	[4]
Shaddock peel	N	35.7%	139 mAh/g at 50 mA/g<br>43 mAh/g at 1000 mA/g	[4]
Bagasse	N	28.1%	142 mAh/g at 100 mA/g	[5]
Bacterial cellulose	N	52%	193 mAh/g at 50 mA/g<br>106 mAh/g at 1000 mA/g	[6]
Cocoon silk	N	-	273 mAh/g at 50 mA/g<br>78 mAh/g at 5000 mA/g	[7]
Sucrose	N	37.7%	301.9 mAh/g at 50 mA/g<br>133.3 mAh/g at 5000 mA/g	[8]
Bamboo	S	50.7%	339.3 mAh/g at 50 mA/g	[9]

charcoal			124.2 mAh/g at 1000 mA/g	
Fucoidan	O	34.1%	392 mAh/g at 50 mA/g<br>107 mAh/g at 10000 mA/g	[10]
Commercial cellulose	O	51%	294 mAh/g at 50 mA/g<br>158 mAh/g at 1000 mA/g	[11]
Water chestnut	O	53.2%	253.2 mAh/g at 100 mA/g<br>134.8 mAh/g at 1000 mA/g	[12]
Rose petal	N/S	-	288.8 mAh/g at 100 mA/g<br>134.6 mAh/g at 1000 mA/g	[13]
Black tea	N/P	-	491.6 mAh/g at 50 mA/g<br>123.4 mAh/g at 1000 mA/g	[14]
Chicken bone	N/P	~66.8%	314 mAh/g at 58 mA/g<br>113 mAh/g at 580 mA/g	[15]
Corn silk	N/O	35.0%	309.8 mAh/g at 100 mA/g<br>105.8 mAh/g at 20000 mA/g	[16]
k-carrageenan	P/S	23.8%	449 mAh/g at 100 mA/g<br>233 mAh/g at 2000 mA/g	[17]
Skimmed cotton	O/S	46.4%	409 mAh/g at 100 mA/g<br>135 mAh/g at 2000 mA/g	[18]
Orange peel	O	63.1%	320.8 mAh/g at 50 mA/g<br>134.6 mAh/g at 2000 mA/g	This work

References

1. Kresse G, Joubert D. From ultrasoft pseudopotentials to the projector augmented-wave method[J]. Physical Review B, 1999, 59(3): 1758.
2. Perdew J P, Burke K, Ernzerhof M. Generalized gradient approximation made simple[J]. Physical Review Letters, 1996, 77(18): 3865.
3. Zhu L F, Zhang Z, Zhang H, et al. Tunable 2D tremella-derived carbon nanosheets with enhanced pseudocapacitance behavior for ultrafast potassium-ion storage[J]. Science China Technological Sciences, 2021, 64(9): 2047-2056.
4. Wang X, Zhao J, Yao D, et al. Bio-derived hierarchically porous heteroatoms doped-carbon as anode for high performance potassium-ion batteries[J]. Journal of Electroanalytical Chemistry, 2020, 871: 114272.
5. Deng Q, Liu H, Zhou Y, et al. N-doped three-dimensional porous carbon materials derived from bagasse biomass as an anode material for K-ion batteries[J]. Journal of Electroanalytical Chemistry, 2021, 899: 115668.
6. Ma L, Li J, Li Z, et al. Ultra-stable potassium ion storage of nitrogen-doped carbon nanofiber derived from bacterial cellulose[J]. Nanomaterials, 2021, 11(5): 1130.
7. Luo H, Chen M, Cao J, et al. Cocoon silk-derived, hierarchically porous carbon as anode for highly robust potassium-ion hybrid capacitors[J]. Nano-micro Letters, 2020, 12(1): 1-13.
8. Qiu Z, Zhao K X, Liu J, et al. Nitrogen-doped mesoporous carbon as an anode material for high performance potassium-ion batteries[J]. Electrochimica Acta, 2020, 340: 135947.
9. Tian S, Guan D, Lu J, et al. Synthesis of the electrochemically stable sulfur-doped bamboo charcoal as the anode material of potassium-ion batteries[J]. Journal of Power Sources, 2020, 448: 227572.
10. Li X, Wang H, Zhang W, et al. High potassium ion storage capacity with long cycling stability of sustainable oxygen-rich carbon nanosheets[J]. Nanoscale, 2021, 13(4): 2389-2398.
11. Nanjundan A K, Gaddam R R, Farokh Niaei A H, et al. Potassium-ion storage in cellulose-derived hard carbon: the role of functional groups[J]. Batteries & Supercaps, 2020, 3(9): 953-960.
12. Xu Z, Du S, Yi Z, et al. Water chestnut-derived slope-dominated carbon as a

high-performance anode for high-safety potassium-ion batteries[J]. ACS Applied Energy Materials, 2020, 3(11): 11410-11417.

13. Hu Z, Liu Z, Zhao J, et al. Rose-petals-derived hemispherical micropapillae carbon with cuticular folds for super potassium storage[J]. Electrochimica Acta, 2021, 368: 137629.
14. Gao Y, Ru Q, Zheng M, et al. Recovery of kitchen bio-waste from spent black tea as hierarchical biomorphic carbon electrodes for ultra-long lifespan potassium-ion storage[J]. Applied Surface Science, 2021, 555: 149675.
15. Yuan X, Zhu B, Feng J, et al. Biomass bone-derived, N/P-doped hierarchical hard carbon for high-energy potassium-ion batteries[J]. Materials Research Bulletin, 2021, 139: 111282.
16. Zhang Y, Zhao R, Li Y, et al. Potassium-ion batteries with novel N, O enriched corn silk-derived carbon as anode exhibiting excellent rate performance[J]. Journal of Power Sources, 2021, 481: 228644.
17. Wei W, Zheng Y, Huang M, et al. A new strategy for achieving high K+ storage capacity with fast kinetics: realizing covalent sulfur-rich carbon by phosphorous doping[J]. Nanoscale, 2021, 13:4911-4920.
18. Xu B, Qi S, Li F, et al. Cotton-derived oxygen/sulfur co-doped hard carbon as advanced anode material for potassium-ion batteries[J]. Chinese Chemical Letters, 2020, 31(1): 217-222.
